# The CCL17/CCL22–CCR4 Axis in Pain Pathogenesis: A Comprehensive Review of Immune-Mediated Mechanisms and Therapeutic Opportunities

**DOI:** 10.1007/s12035-025-05550-9

**Published:** 2025-11-26

**Authors:** Amin Hasheminia, Sarah Mann, Kiera Liblik, Mohammad El-Diasty

**Affiliations:** 1 Faculty of Medicine and Health Sciences, McGill University, Montreal, QC Canada; 2 Department of Biomedical and Molecular Sciences, Queen’s University, Kingston, ON Canada; 3 Department of Medicine, Queen’s University, Kingston, ON Canada; 4 Division of Cardiac Surgery, Harrington Heart and Vascular Institute, University Hospitals Cleveland Medical Centre, Cleveland, OH 44106 USA

**Keywords:** CCL17/CCL22–CCR4 axis, Pain pathogenesis, Immune-mediated mechanisms and therapeutic opportunities

## Abstract

The role of C–C motif chemokine ligand (CCL) 17 and CCL22 signalling has been demonstrated in allergic disorders, such as asthma and atopic dermatitis, as well as multiple types of neoplastic disorders. New evidence has identified that CCL17/CCL22 activation of the receptor CCR4 functions to mediate pain, with distinct roles in arthritic, neuropathic, and inflammatory postoperative pain. CCR4 blockade is suggested to prevent the development of neuropathic pain by inhibiting microglia activation and the subsequent increase in pronociceptive cytokines. CCL17 can also play a key role in the pathophysiology of arthritic pain. Further, CCL17 expression is increased in response to granulocyte–macrophage colony-stimulating factor. Both CCL17 and CCL22, produced by skin-resident dendritic cells in response to incisional wounds, bind CCR4 expressed on peripheral sensory neurons, directly inducing pain signalling. These findings suggest that CCR4 may represent a therapeutic target for reducing pain. Understanding the role of CCR4 in the process of pain may also provide essential insight into the development of these therapeutic agents. The present paper provides a comprehensive review of the current literature on the role of the immune system in the inflammatory pain response, with a specific focus on the role of CCL17 and CCL22 activity in the pathophysiology of pain. Also, we discuss the potential for therapeutically targeting CCR4 and its clinical implications.

## Introduction

The immune system is closely involved in the development and maintenance of pain, predominantly through the action of immune mediators on sensory neurons [1]. Specifically, cytokines released in response to noxious stimuli act on nociceptive neurons, stimulating the sensation of pain [1]. Chemokines are a type of cytokine typically involved in cell migration, though they have also been implicated in pain signalling. The C–C motif chemokine ligands (CCL) CCL17 and CCL22 are of particular interest, referred to together as CCL17/22 [2]. CCL17 and CCL22, previously called thymus and activation-regulated chemokine and macrophage-derived chemokine, respectively, are both agonists of the CCR4 receptor. These chemokines are primarily known for inducing chemotaxis of type 2 helper T cells (Th2) [3]. Through this mechanism, CCL17/22 may contribute to numerous disease processes, including the pathogenesis of atopic dermatitis [4], asthma [5], and multiple oncological disorders [6]. Current evidence suggests that CCL17/22 can contribute to pain through an alternative mechanism, although further investigation is required to elucidate if CCL17/22-mediated cell migration plays an additional role in pain signalling. Identifying a role for CCR4 activation in pain may aid in developing associated therapeutic agents for pain alleviation. Accordingly, a comprehensive understanding of CCL17/22 signalling via CCR4 is necessary to develop effective treatments that target CCR4 and/or its ligands. The present narrative review aims to synthesize the current literature on the roles of CCL17, CCL22, and CCR4 in the pathogenesis of pain and the underlying signalling mechanisms.

## The Neuroimmune Component of Pain

### Pain Development

Nociceptors are sensory neurons whose axons are primarily classified as thinly myelinated Aδ fibers or unmyelinated C fibers [1]. Pain arises from action potentials generated by nociceptors in response to chemical, mechanical, or thermal stimuli [7]. These signals propagate via dorsal root ganglion (DRG) neurons to the brain for processing as pain [7]. Immune mediators like chemokines can activate nociceptors, which release neuropeptides to modulate immune activity [7]. Chemokine-induced peripheral sensitization lowers the nociceptor activation threshold, increasing pain sensitivity and contributing to chronic pain development, a significant area of research due to the global chronic pain burden [1, 8]. Similarly, DRG neurons can undergo central sensitization, where reduced primary nociceptor input heightens pain sensitivity in non-inflamed areas [9]. This inflammation–pain interaction underpins the use of non-steroidal anti-inflammatory drugs (NSAIDs) as common pain treatments [1, 8].

### Modulation of Nociceptor Activity By Cytokines

Tumour necrosis factor (TNF) and interleukin (IL)−1β are proinflammatory cytokines previously established as contributing to the induction of pain. Both TNF-α and IL-1β can sensitize nociceptors by mediating the phosphorylation of sodium channels, which facilitates channel opening and depolarisation of the neuron, thus initiating the pain signalling process [1, 9, 10]. Moreover, binding of these cytokines to their respective receptors promotes the release of prostaglandins [1, 9, 10]. These pathways can lead to increased neuronal firing and hyperalgesia, the exaggerated pain response to noxious stimuli. However, in chronic pain, the IL-1 receptor antagonist is downregulated, allowing IL-1β to act with limited inhibition [11].

In addition to cytokines, bradykinin is believed to play a pivotal role in modulating nociceptor activity during inflammation. Bradykinin sensitizes nociceptors by activating its receptors, B1 and B2, which are upregulated in inflammatory conditions [12]. This activation leads to the production of secondary messengers such as prostaglandins and nitric oxide, which amplify nociceptive signalling [13]. Furthermore, bradykinin has been shown to increase the expression and activity of TRPV1 channels, contributing to thermal hyperalgesia and chronic pain [12]. The combined actions of cytokines like TNF and IL-1β, along with bradykinin, underscore their central role in driving the transition from acute to chronic pain states.

### Immune System Response to Pain Signalling

Nociceptors influence immune cell activity through neurotransmitter and neuropeptide release, such as calcitonin gene-related peptide (CGRP) and substance P (SP). CGRP generally promotes anti-inflammatory responses by upregulating IL-10, which downregulates nociceptor sodium channels, and inhibits the production of pro-inflammatory cytokines like IL-12, IL-6, and TNF-α from dendritic cells [1, 8, 9]. In contrast, SP increases pro-inflammatory mediators, inducing macrophages and monocytes to release TNF-α, IL-1, and IL-6, while also enhancing mast cell degranulation to release nerve growth factor (NGF), which sensitizes nociceptors [1, 14]. Pain signalling is further amplified by glial activation and immune cell infiltration. Microglia in the central nervous system (CNS) play a critical role in modulating pain. Upon activation, microglia release brain-derived neurotrophic factor (BDNF), IL-1β, and IL-6, which bind to dorsal root ganglion (DRG) neurons, driving central sensitization [1, 9]. These inflammatory mediators also promote the recruitment of neutrophils into the DRG, where they secrete cytokines and chemokines that sustain immune cell infiltration and amplify the pain response [14]. The infiltration of neutrophils and subsequent cytokine release create a feedforward loop, exacerbating pain signalling and contributing to chronic pain development.

## Contribution of Chemokines to Pain Responses

Chemokines are small proteins categorised into four groups based on the location of two cysteine residues near the N-terminus [15]. These categories include CC, CXC, CX3C, and XC, with X representing any amino acid [15]. Each group has corresponding receptors that follow the same naming system. Chemokines are well known for driving cell migration, particularly chemotaxis of leukocytes. Additionally, immune response, embryonic development, and homeostasis are all influenced by chemokine activity [15]. Several chemokines have been identified as mediators of the pain response, with distinct roles in neuropathic and inflammatory pain. The chemokines currently known to contribute to pain pathogenesis include CCL2, CCL3, CCL7, CXCL1, CXCL10, CXCL12, CXCL13, and CX3CL1 [16].

### Inflammatory Pain

**Fig. 1:**
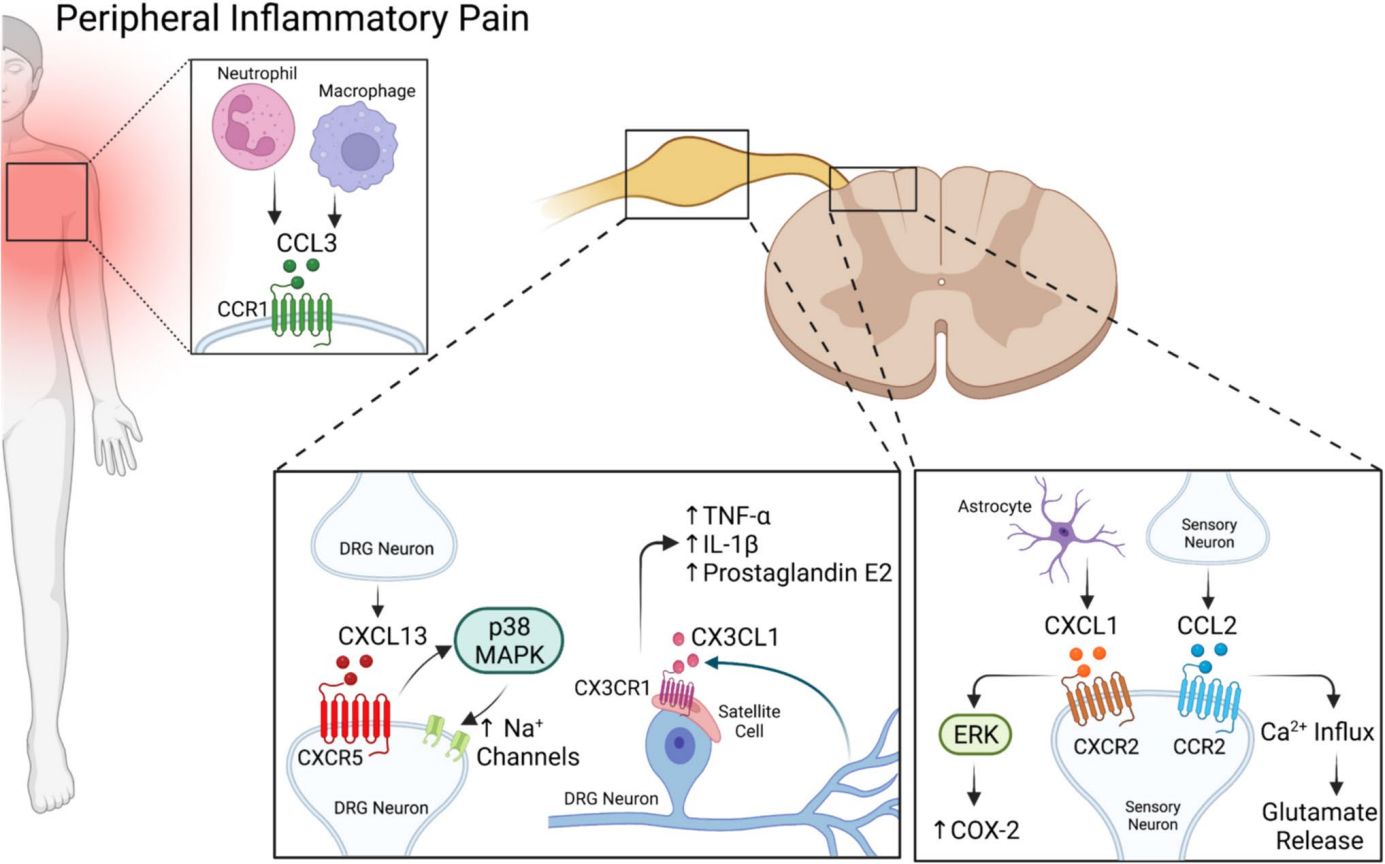
Overview of the chemokines upregulated during inflammatory pain and the outcomes of chemokine receptor activation. In inflamed areas, neutrophils and macrophages upregulate production of CCL3. CXCL13 is upregulated by DRG neurons and binds CXCR5, resulting in increased Na^+^ channel density. DRG neurons also secrete more CX3CL1, which binds CX3CR1 expressed on satellite cells. Activation of CX3CR1 results in increased levels of TNF-α, IL-1β, and prostaglandin E2. In the dorsal horn of the spinal cord, sensory neurons upregulate CXCR2 and CCR2, the receptors for CXCL1 and CCL2, respectively. Astrocytes increase production of CXCL1, leading to increased COX-2. CCL2 binding CCR2 results in glutamate release. Created with BioRender.com

As illustrated in (Fig. 1), in inflamed tissue, CCL2, CXCL1, CXCL13, CX₃CL1, and CCL3 accumulate around the dorsal horn. CCL2 binding CCR2 quickly raises presynaptic calcium, CXCL1 at CXCR2 triggers ERK and cyclo-oxygenase-2, CXCL13 engaging CXCR5 uses p38 MAPK to boost sodium-channel density, CX₃CL1 acting on CX₃CR1 stimulates the release of TNF-α and IL-1β, and CCL3 signalling through CCR1 or CCR5 can either sustain or resolve hyperalgesia [17–22]. Table 1 lists the cellular sources, signalling cascades, and behavioural outcomes for each of these pathways.

**Table 1:** Inflammatory-pain chemokine pathways

Chemokine → Receptor	Source → Target	Key signals	Functional outcome
CCL2 → CCR2 [17]	Macrophage or keratinocyte → presynaptic dorsal-horn neuron	CaV2.2 Ca^2^⁺ influx, pERK, PKC	Early central sensitisation, lowered withdrawal thresholds
CXCL1 → CXCR2 [18]	Lumbar astrocyte → postsynaptic neuron	ERK1/2, COX-2, PGE₂	Persistent heat and mechanical hyperalgesia
CXCL13 → CXCR5[19]	DRG neuron → adjacent neuron	p38 MAPK, Nav1.8 up-regulation	Increased afferent firing and ectopic activity
CX₃CL1 → CX₃CR1[20]	Cleaved neuronal CX₃CL1 → satellite glia	p38 MAPK, NF-κB	Glial TNF-α, IL-1β release and amplified sensitisation
CCL3 → CCR1/CCR5 [21, 22]	Neutrophil or macrophage → nociceptor and glia	PLCβ-IP₃-Ca^2^⁺, NF-κB	Hyperalgesia reversible with anti-CCL3 or dual CCR1/5 blockade

### Neuropathic Pain

**Fig. 2:**
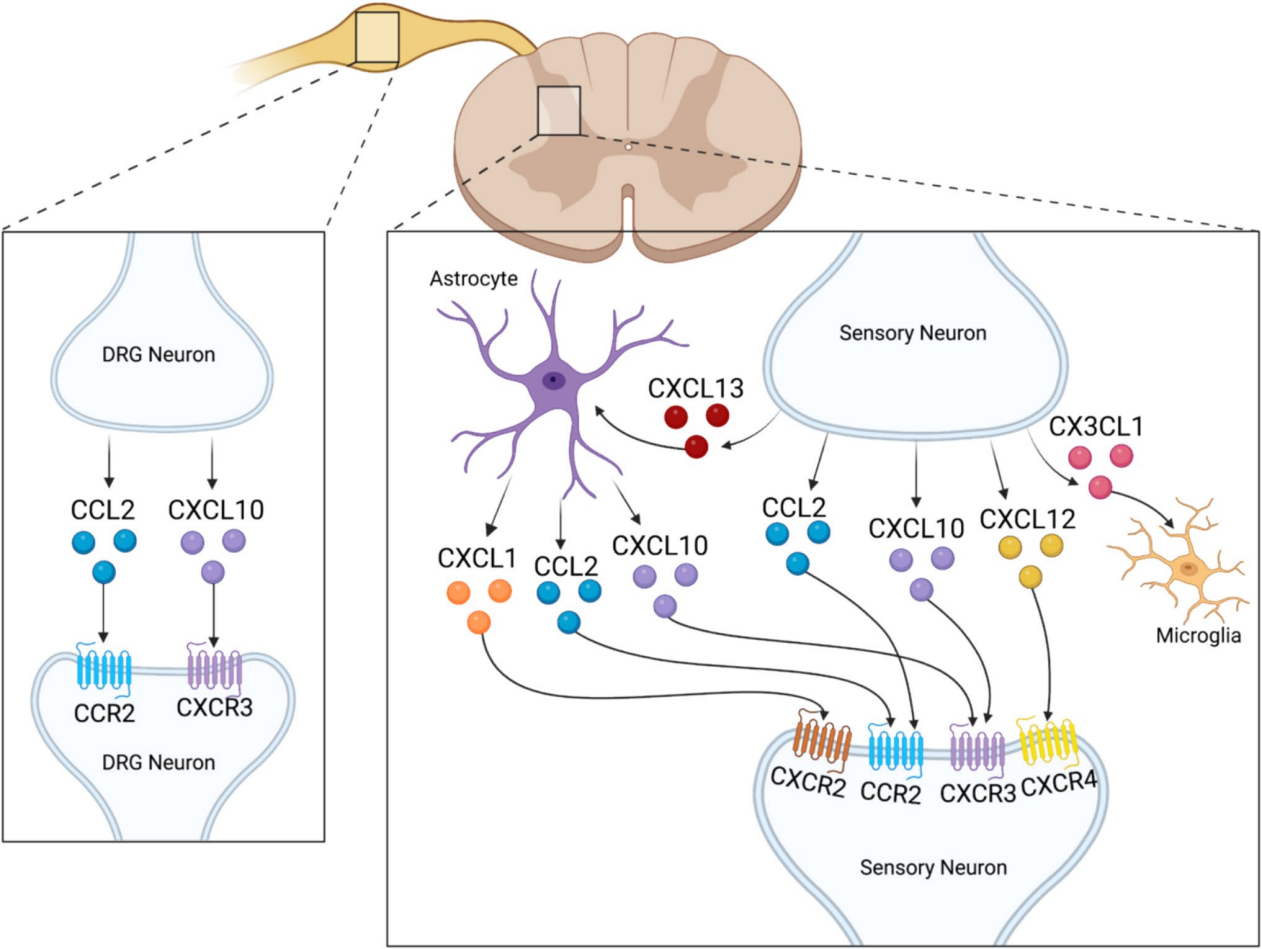
Overview of the chemokines upregulated in models of neuropathic pain. In the spinal cord, sensory neurons increase the production of CXCL13, CCL2, CXCL10, CXCL12, and CX3CL1. In addition, the chemokine receptors CXCR2, CCR2, CXCR3, and CXCR4 are upregulated. Astrocytes in the spinal cord secrete increased CXCL1, CCL2, and CXCL10. In the dorsal root ganglion, neurons upregulate CCL2 and CXCL10, and the receptors CCR2 and CXCR3. Created with BioRender.com

After nerve injury, the same chemokines reappear in new compartments. Figure 2 shows that CXCL10, CCL2, and CCL7 surge in dorsal-root-ganglion neurons, astrocytes become rich sources of CXCL1, CXCL10, and CXCL12, soluble CX₃CL1 activates microglial CX₃CR1, and CXCL13 becomes the dominant spinal chemokine, acting on astrocytic CXCR5 in diabetic neuropathy and on neuronal CXCR5 in post-ischaemic pain [23–35]. Table 2 expands these neuropathic circuits, detailing how each ligand–receptor pair drives microglial activation, cytokine release, or persistent neuronal excitability that sustains mechanical and thermal hypersensitivity.

**Table 2:** Neuropathic-pain chemokine pathways

Chemokine → Receptor	Model phase and compartment	Signalling cascade	Behavioural effect
CXCL10 → CXCR3 [23]	Early chronic-constriction injury, astrocyte → neuron	JNK, CREB, GluA1 trafficking	Elevated EPSC frequency, tactile allodynia
CCL2/CCL7 → CCR2 [24]	Early chronic-constriction injury, neuron or astrocyte → microglia	pERK, p38, IL-1β	Maintained mechanical hypersensitivity
TNF-α-induced CCL2 → CCR2 [25]	Peripheral-nerve injury, Schwann cell → microglia	ERK, PI3K, ROS	Ongoing pain blocked by CCR2 or TNF inhibition
Soluble CX₃CL1 → CX₃CR1[26]	Peripheral-nerve injury, neuron → microglia	p38, BDNF, KCC2 down-regulation	Robust mechanical allodynia
CCR2- or CX₃CR1-deficiency [27, 28]	Peripheral-nerve injury	Microgliosis reduced	No mechanical allodynia despite nerve damage
CXCL1 → CXCR2 [29]	Maintenance after spinal-nerve ligation, astrocyte → neuron	Sustained ERK, COX-2	Prolonged hyperalgesia that resolves with delayed anti-CXCL1
CXCL9/10/11 → CXCR3 [30]	Spinal-nerve ligation, astrocyte → neuron	CXCL10 drives pJNK and c-Fos	CXCL10 neutralisation reverses pain
CXCL12 → CXCR4 [31, 32]	Spinal-nerve ligation, neuron or astrocyte → CXCR4	CXCR4–TLR4 loop, STAT3	Escalating TNF-α, IL-1β, IL-6; CXCR4 blockade normalises thresholds
CXCL13 → CXCR5 [33]	Spinal-nerve ligation, neuron → astrocyte	Astrocytic p38, NLRP3	IL-1β maturation, central sensitisation
CXCL13 → CXCR5 (astrocytic) [34]	Painful-diabetic neuropathy	pERK, pSTAT3, pAKT	IL-1β, TNF-α, IL-6 rise, long-lasting allodynia
CXCL13 → CXCR5 (neuronal) [35]	Chronic post-ischaemic pain	NF-κB, IL-6 up-regulation	Sustained tactile allodynia

## Expression and Regulation of CCL17, CCL22, and CCR4

### Identification of CCL17 Expression

The first study characterising CCL17 found it was constitutively expressed in the thymus, and expression could be induced in peripheral blood mononuclear cells when stimulated by phytohemagglutinin [36]. The study showed that CCL17 could bind peripheral T cells. It mapped the gene encoding CCL17 to chromosome 16 [36]. Subsequent studies identified CCR4 as the receptor for CCL17, expressed on CD4+ human T cell lines and peripheral blood T cells [37]. CCL17 binding to CCR4 induces chemotaxis of T cells and intracellular calcium influx [36, 37]. CCL17 has two distinct binding sites that are required for normal function, as blocking either site with a monoclonal antibody can inhibit its function in both in vitro and in vivo models [38]. Monocytes and monocyte-derived DCs were later identified as a source for CCL17 when stimulated by granulocyte–macrophage colony-stimulating factor (GM-CSF), IL-3, and IL-4 (3). CCL17 production by murine Langerhans cells is promoted by TNF-α, transforming growth factor β (TGF-β1), IL-4, and IL-13, and inhibited by GM-CSF, IFN-γ, IL-10, and LPS [39].

### Identification of CCL22 Expression

When CCL22 was first identified, it was found to be highly expressed in the healthy thymus, macrophages, and monocyte-derived DCs, and it induces migration of activated natural killer cells, monocyte-derived DCs, and monocytes [40]. However, some literature suggests that CCL22 may not effectively attract monocytes [3]. CCL22, like CCL17, is encoded on chromosome 16 and has two binding sites required for function [38, 40].

IL-4, IL-13, LPS, and TNF-α stimulate CCL22 expression in macrophages [41]. Further, CCL22 production by murine Langerhans cells is promoted by TNF-α, GM-CSF, IL-1β, and IL-4, and inhibited by IFN-γ and IL-10 [39]. Under homeostatic conditions, the majority of CCL22 is found in the thymus and lymph nodes [42]. In the lymph nodes, only CD11c^+^DCs can produce CCL22 (38). CCL22 expression is required to promote cell contact between the DCs and CCR4+ regulatory T cells (Tregs) [42]. In mice deficient in CCL22, effector T cells make the majority of contact with DCs, resulting in enhanced adaptive immune responses [42]. Constitutive expression of CCL22 in DCs depends on GM-CSF, which is produced by T cells [43].

### CCR4 Expression and Response to Agonist Binding

CCR4 is predominantly expressed on Th2 cells [3], as well as multiple T cell subsets, including systemic memory CD4+ T cells [44], skin-homing T cells that express cutaneous lymphocyte antigen (CLA) [44], Tregs [45], Th17 cells [46], Th22 cells [47], and a CD4 natural killer T cell subset [48]. Additionally, CCR4 expression has been noted on IL-2 activated NK cells [49], airway eosinophils and epithelial cells [50, 51], platelets [52], and peripheral sensory neurons [2]. In the CNS, low levels of CCR4 have been reported in astrocytes and the CHEM3 microglia cell line [53], and CCR4 expressed on microglia in the nucleus accumbens is upregulated by morphine treatment [54].

CCL17/22 bind the same orthosteric site on CCR4 [55]. CCL22 is the more potent agonist, causing rapid receptor internalization upon binding, which prevents further chemotactic response to either ligand. In contrast, receptor internalization is less pronounced in response to CCL17, and CCL22 can still initiate chemotaxis after CCL17 binding [55, 56]. Identification of two distinct CCR4 conformations, one binding both ligands and the other binding only CCL22, has been suggested as the explanation for the continued response to CCL22 after CCL17 stimulation [57]. CCR4 has two distinct allosteric binding sites [55]. Site one binds lipophilic amines, which induces receptor internalization, while site two binds arylsulphonamides, and no receptor internalization is seen [55]. Both types of antagonists prevent CCL17/22 binding and inhibit chemotaxis [55].

### Signalling Mechanisms Mediated by CCR4 Activation

CCR4, along with all CC chemokine receptors, is a G protein-coupled receptor (GPCR) [58]. Structurally, GPCRs include seven transmembrane α-helices, an extracellular N-terminus, and an intracellular C-terminus [58]. Conflicting results have been published regarding whether activated CCR4 signals via G proteins or β-arrestin. Accordingly, Ajram et al. (2014) reported that only CCL22 induces coupling of CCR4 to β-arrestin [55], while Lim et al. (2008) showed β-arrestin recruitment to CCR4 in response to both ligands. Still, a significantly stronger response to CCL22 [59] was found by Lim et al. (2008), who reported no evidence of G-protein mediated signalling in response to CCL17 or CCL22 when using intact Chinese hamster ovary (CHO) cells [59]. In contrast, Ajram et al. (2014) found both ligands induce G-protein binding to the isolated membranes of CHO cells, with a stronger response seen from CCL22 [55]. Moreover, treatment of HUT78 cells with pertussis toxin to inhibit G-proteins prevents chemotaxis in response to CCL17/22 [55]. In Th2 cells from wildtype and β-arrestin-2 knockout mice, treatment with pertussis toxin prevented chemotaxis in response to CCL22 [60]. These studies indicate that G-proteins are required for chemotaxis of leukocytes. Calcium mobilisation is also generally accepted to be mediated by G-proteins and has been observed numerous times following CCR4 activation [37, 57, 61]. The difference in results reported by Lim et al. (2008) and the described studies could be due to variations in cell line, cell preparation, and assay choice. Murine Th2 cells lacking β-arrestin-2 have an impaired chemotactic response to CCL22, but no difference in response to CCL17, suggesting CCL22 promotes β-arrestin-2-dependent chemotaxis [60].

### Overview of CCL17/CCL22–CCR4 Signalling via CXCR4

In addition to its role in CCR4-mediated signalling, CCL17 and CCL22 have been implicated in distinct physiological and pathophysiological processes through their interaction with CXCR4 [62]. Upon binding with CXCR4, these chemokines trigger downstream signalling pathways that are critical for chemotaxis and modulating cellular function in diverse contexts [62]. CCL17- or CCL22-induced CXCR4 activation leads to a cascade involving the activation of PI3K, Akt, and ERK pathways, which are crucial for cell survival, proliferation, and migration [62, 63]. In immune cells, this interaction facilitates chemotaxis by regulating cytoskeletal reorganisation and promoting directional cell movement toward higher chemokine gradients [64, 65].

In pathophysiological conditions, such as cancer metastasis, the CCL17/CCL22–CXCR4 axis enhances tumour cell migration and invasion by modulating the tumour microenvironment [64, 66]. These chemokines attract regulatory T cells (Tregs) and other immunosuppressive cell populations to the tumour site, which aids in immune evasion [65]. Additionally, in chronic inflammatory diseases, such as rheumatoid arthritis, the signalling mediated by CCL17 and CCL22 via CXCR4 exacerbates inflammatory cell recruitment and tissue damage [62, 67].

Conversely, under normal physiological conditions, this signalling axis supports tissue homeostasis. For example, it contributes to the resolution of inflammation by recruiting anti-inflammatory cells and modulating macrophage polarisation toward an M2 phenotype [68]. This dual role highlights the context-dependent nature of CCL17/CCL22 signalling through CXCR4, which balances immune activation and resolution to maintain cellular and systemic equilibrium.

## The Role of CCL17 and CCL22 in Other Clinical Conditions

### Bronchial Asthma

CCL17 and CCL22 are now recognised as central drivers of allergic asthma, orchestrating the recruitment of CCR4-positive Th2 cells that sustain type-2 airway inflammation. Table 3 gives an at-a-glance view of the human and animal evidence, beginning with the consistent elevation of CCL17 in serum and sputum of asthmatic patients [69, 70] and the post-challenge surge of both ligands in broncho-alveolar lavage fluid [60]. These chemokines guide Th2 chemotaxis [71], and the number of CCR4^+^ T cells in sputum or blood rises in proportion to clinical severity [72]. In mice, dendritic cells release CCL17 and CCL22 after allergen exposure, a response amplified by nitric oxide and later damped by TGF-β [73]. Neutralising CCL17 lowers eosinophil counts yet unexpectedly increases airway resistance [74], while complete genetic loss of CCR4 leaves lung mechanics largely unchanged [75]. Even so, a small-molecule CCR4 antagonist markedly reduces airway inflammation in a humanised mouse model [5].

**Table 3:** CCL17/CCL22 in bronchial asthma

Observation or intervention	Model or cohort	Source → target	Key outcome
Serum and sputum CCL17 above control range [69, 70]	Adult asthma out-patients	Blood monocytes/airway DCs → circulating Th2 cells	Median serum 145 pg mL⁻^1^ vs 40 pg mL⁻^1^ in controls
BAL CCL17/22 surge after allergen challenge [60]	Allergen-challenge volunteers	Airway DCs → eosinophils	Five-fold ligand rise at 24 h
Th2 chemotaxis via CCR4 sustains disease [71]	Integrative review	CCL17/22 → CCR4^+^ IL-4/IL-13 Th2 cells	Drives mucus production and hyper-responsiveness
CCR4^+^ sputum T cells correlate with severity [72]	36 asthmatic adults	CCR4^+^ CD4 T cells → airway epithelium	*r* = 0.53 with FEV₁ reduction
DC subsets modulate ligands via NO and TGF-β [73]	OVA mouse model	CD11b⁺NO^high^ vs CD11b⁻TGF-β^high^ DCs	Peak CCL17/22 at 36 h, suppression at 60 h
Anti-CCL17 lowers eosinophils, raises resistance [74]	Chronic OVA model	Neutralising Ab → lung	45% fewer eosinophils, 30% ↑ airway resistance
CCR4 knock-out shows no benefit [75]	CCR4⁻/⁻ mice	Global deletion	Eosinophils and resistance unchanged
Small-molecule CCR4 blocker curbs inflammation [5]	Humanised dust-mite model	Drug 3 mg kg⁻^1^ → lung	55% fewer eosinophils, 50% fewer CD3 T cells

### Atopic and Contact Dermatitis

In atopic dermatitis, the same CCR4 axis shapes cutaneous inflammation, though its impact varies with genetic background. Table 4 compiles the clinical and experimental data, starting with the tight correlation between serum CCL17 and disease severity across patient cohorts [76–78] and extending to the multilinage expression of the ligand within lesional skin [79]. House-dust-mite antigens and the cytokines IL-22, TNF-α, and IFN-γ further boost keratinocyte CCL17 production [80, 81]. Functional studies diverge: C57BL/6 mice lacking CCR4 remain susceptible to dermatitis [82, 83], while BALB/c knock-outs are protected, and topical Compound 22 in this strain curtails Th2 and eosinophil influx [4, 84]. The vitamin-D analogue MC903 model confirms that CCR4 draws both Th2 and Th17 cells, and its blockade normalises epidermal thickness [85]. An oral antagonist, RPT193, produced rapid, transcriptome-linked improvement in moderate-to-severe disease [86], and genetic loss of CCL22 itself eases allergic contact dermatitis [87].

**Table 4:** CCL17/CCL22 in skin inflammation

Observation or intervention	Model/cohort	Source → target	Main effect
Serum CCL17 correlates with SCORAD [76–78]	120 AD patients	Blood DCs → CCR4^+^ skin-homing T cells	*r* = 0.69 with index
Multicellular CCL17 expression in lesions [79]	Patient biopsies	Keratinocyte, DC, endothelium	Dense ligand staining in basal layer
HDM and cytokines boost keratinocyte CCL17 [80, 81]	HaCaT culture	IL-22R, TNF-α, IFN-γ → CCL17	Synergistic induction
CCR4 loss fails to protect C57BL/6 mice [82, 83]	DNFB dermatitis	Global knock-out	Lesion scores unchanged
CCR4 required in BALB/c strain [4]	Oxazolone model	Gene deletion	40% less ear swelling
Topical Compound 22 reduces lesions [84]	BALB/c, 30 µM	Drug → skin	60% fewer Th2, milder pathology
CCR4 drives Th2/Th17 influx in MC903 model [85]	Vitamin-D analogue dermatitis	Compound 22 systemic	Epidermis 55% thinner
Oral RPT193 improves moderate-severe AD [86]	Phase 1 trial	Drug 800 mg day⁻^1^	−52% EASI day 29
CCL22 knock-out eases contact dermatitis [87]	TNCB CHS model	Gene deletion	Ear swelling − 60%

### Neoplastic Disorders

CCL17 and CCL22 also shape anti-tumour immunity, generally to the host’s detriment. Table 5 summarises how high intratumoural levels of either chemokine recruit CCR4-positive regulatory T cells and predict poorer survival in ovarian and breast cancers [88–90]. Tumour-secreted IL-1 can trigger dendritic-cell CCL22 release, an effect blocked by an IL-1 receptor antagonist [91]. In a pancreatic-cancer model, pharmacological CCR4 antagonism lowers Treg density and slows tumour growth [92]. Not every setting is identical, because abundant CCL22 predicts better survival in tongue and mouth-floor squamous carcinoma, perhaps through preferential attraction of Th2 rather than regulatory cells [93]. High CCR4 expression also links the pathway to adult Tcell leukaemia–lymphoma and cutaneous T cell lymphoma [6].

**Table 5:** CCL17/CCL22 in tumour immunity

Observation or intervention	Tumour type	Source → responder	Outcome
Ligands recruit CCR4^+^ Tregs, shorten survival [88–90]	Ovarian, breast	Tumour DCs → FoxP3 Tregs	Shorter DFS and OS
Tumour IL-1 drives DC CCL22; IL-1R block halts it [91]	Pancreatic adenocarcinoma culture	Supernatant → monocyte DCs	CCL22 down 75% with IL-1RA
CCR4 antagonist lowers Tregs and slows tumour [92]	Mouse pancreatic cancer	Drug CCR4-351 → tumour	60% fewer GFP-Tregs
High CCL22 links to better outcome [93]	Tongue/mouth-floor SCC	Tumour epithelium → Th2 cells	Five-year OS 70% vs 42%
CCR4 high in ATLL and CTCL [6]	T cell malignancies	Malignant T cell autocrine loop	Supports tumour survival

## CCL17 and CCL22 in Pain Pathogenesis

### CCL17 and CCL22 Administration Induces Pain Responses

Initial evidence suggesting that CCL17/22 contribute to pain was reported by Oh et al. (2001), who observed CCR4 expression on rat DRG neurons [94]. In addition, they found that most of the neurons expressing CCR4 also expressed SP and capsaicin receptors (TRPV1), which identified the neurons as nociceptors. CCL17/22 applied to isolated DRG neurons caused an increase in intracellular calcium, indicating neuronal excitation. Moreover, this study showed that intradermal injection of CCL22 resulted in tactile allodynia [94].

Numerous studies have subsequently demonstrated pain in response to CCL17/22 administration. Intraplantar or intrathecal injection of CCL17 induces inflammatory pain, with pain measured by changes in weight distribution [95–98], and the development of mechanical and thermal hypersensitivity [2, 99, 100]. However, the reported responses to CCL22 administration have been inconsistent. After intraplantar CCL22 injection, Lee et al. (2020) reported no change in weight distribution [98], while Silva et al. (2022) showed significant mechanical and thermal hypersensitivity [2]. Rats given 0.92 nmol/5 µl CCL22 per day did not show thermal hyperalgesia [101]. This lack of pain response could be merely due to the low dose, as Bogacka et al. (2020) reported that 0.615 nmol/5 µl CCL22 was able to induce mechanical and thermal hypersensitivity [99, 100].

Despite these findings, recent data challenge the notion of CCR4 expression on DRG neurons. Cook et al. (2018) conducted RNA sequencing (RNA-seq) on purified DRG neurons isolated from mice and found no detectable expression of CCR4 transcripts [10]. This result contrasts with earlier findings and raises questions about the source of CCR4 expression reported by Oh et al. (2001) [94]. Cook et al. (2018) hypothesise that contamination from non-neuronal cells, such as resident macrophages or Schwann cells, in earlier preparations may have contributed to the detection of CCR4. Given that non-neuronal cells are known to express CCR4, this alternative explanation warrants consideration [10].

Clinically, elevated CCL17/22 levels have been recorded in multiple diseases associated with chronic pain, including chronic prostatitis/chronic pelvic pain syndrome and fibromyalgia [102]. Patients with acute myofascial pain have higher levels of CCL22 [103], and in atopic dermatitis, increasing serum CCL17 correlates with increasing pain [104]. Various animal models have also reported that CCL17 and CCL22 may be related to pain pathogenesis. In their study, Ren et al. reported a positive correlation between CCL22 and pain after joint injury in rats [105]. Similarly, it was found that CCL17-deficient mice with induced arthritis did not develop pain after joint injury, suggesting that CCL17 is required for the genesis of arthritis pain [95, 96, 98, 106, 107].

### The Role of CCL17 in Arthritic Pain

**Fig. 3:**
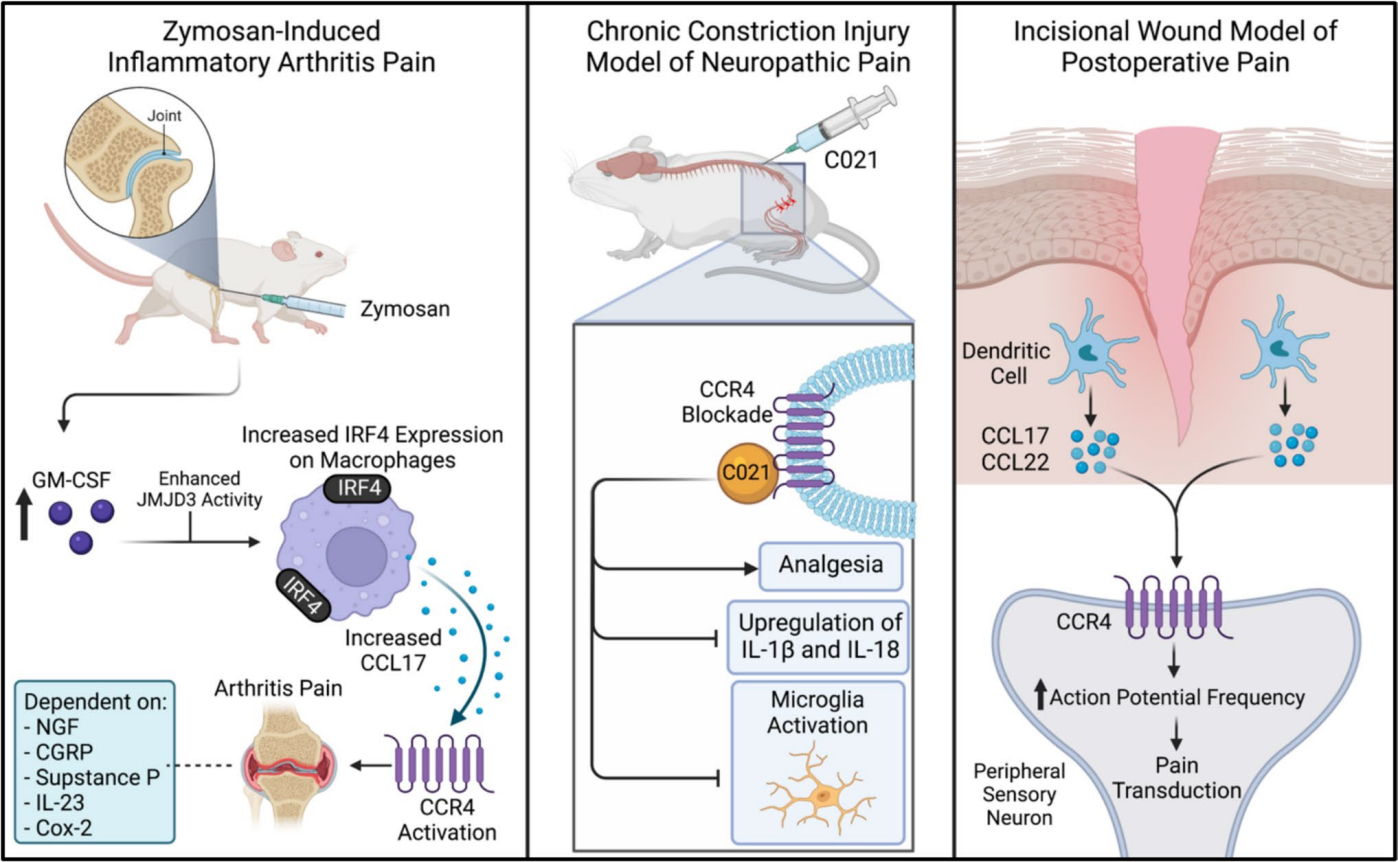
In zymosan-induced inflammatory arthritis, pain is driven by an increase in GM-CSF. This enhances JMJD3 demethylase activity, which increases expression of IRF4 in macrophages. Macrophages then produce CCL17, which binds CCR4, leading to arthritis pain. Downstream of CCL17, arthritis pain is dependent on NGF, CGRP, substance P, IL-23, and COX-2. In the chronic constriction injury model of neuropathic pain, administration of the CCR4 antagonist C021 induces analgesia and prevents activation of microglia and upregulation of pronociceptive cytokines IL-1β and IL-18. In the incisional wound model of postoperative pain, dendritic cells in the epidermis produce CCL17 and CCL22, which act directly on CCR4 expressed on peripheral sensory neurons to increase action potential frequency and initiate pain signalling. Created with BioRender.com

Evidence of CCL17 contributing to pain responses via CCR4 predominantly comes from murine models of arthritis, demonstrating that CCL17 can drive arthritic pain and may contribute significantly to the development of chronic pain (Fig. 3) [95, 96, 98, 106, 107]. Further, it has been delineated that inflammatory and arthritic pain driven by GM-CSF requires downstream CCL17 activity [95]. Further, current literature suggests that GM-CSF enhances Jumonji domain-containing protein D3 (JMJD3) demethylase activity, which upregulates the expression of IFN regulatory factor 4 (IRF4) on human monocytes and murine macrophages [95]. IRF4 then mediates the increased CCL17 production [95]. When monoarticular arthritis is modelled by injecting mBSA into the knee joint, followed by CCL17 into the neck of GM-CSF knockout mice, pain responses are observed. This suggests CCL17-driven pain does not require GM-CSF or IRF4. GM-CSF is upstream from CCL17 [95]. Downstream, CCL17-driven inflammatory pain is dependent on COX2 [95], IL-23 [97], NGF, CGRP, and SP [98]. Interestingly, Achuthan et al. observed CCL17-driven monoarticular arthritis pain in RAG1 knockout mice, suggesting that pain may be mediated by T or B cell activity [95]. Further, Cook et al. (2018) found that TNF-α is required for activation of the GM-CSF/CCL17 signalling pathway, indicated by the lack of pain and CCL17 upregulation following prophylactic blockade of TNF-α [96]. Once pain has been established, this suggests that TNF-α is not required for CCL17 production [96].

Furthermore, recent studies have broadened the role of CCL17 in osteoarthritis to include obesity-associated pain and disease. Shin et al. [108] demonstrated that the GM-CSF/CCL17/CCR4 pathway plays a critical role in exacerbating OA development in obese murine models [109]. Specifically, obesity-induced knee joint damage was found to be dependent on GM-CSF and CCL17, independent of CCL22. Obesity alone led to spontaneous knee joint damage and elevated pain-like behaviours, reinforcing the pathogenic role of CCL17 in this context [108]. Supporting these findings, clinical data from knee OA patients revealed a correlation between BMI and circulating CCL17 levels, as well as increased GM-CSF and CCL17 gene expression in OA synovial tissue. These observations highlight the potential of targeting GM-CSF and CCL17 for the treatment of obesity-associated OA pain and disease [108].

In collagenase-induced osteoarthritis, CCL17 expression in macrophages requires GM-CSF and IRF4, and CCL17 binding to CCR4 is required for pain [107]. Similarly, in the mouse model of monoarticular arthritis studied by Lee et al. (2020), the pain was determined to be mediated by CCR4 expressed on non-bone marrow-derived cells [98]. As has been seen in similar studies, inflammatory and arthritic pain responses were consistently ameliorated by monoclonal antibodies against CCL17 [95, 96, 106, 107], and were not seen in mice deficient in CCR4 [98, 107].

### The Role of CCR4 in Neuropathic Pain

As with arthritic pain, CCR4 mainly contributes to neuropathic nociception and pain mediation. Two murine studies of neuropathic pain by Bogacka et al. (2020) delineated that CCR4 activation contributes to the development of pain, which can be inhibited with the CCR4 antagonist C021 (Fig. 3) [99, 100]. Interestingly, C021 also attenuated pain driven by CCL2 and inhibited an increase in CCL2 mRNA in the spine, as seen normally after CC [86]. Similar results were reported in a third study by Bogacka et al. (2020) regarding chronic pain in diabetic mice, where CCL2 was upregulated in the spinal cord and C021 attenuated pain responses [109]. This suggests neuropathic pain could be mediated by CCL2, rather than CCL17 or CCL22. However, it has been established that CCL17/22 are upregulated in the DRG after CCI [100]. An increase in IBA1 and GFAP, biomarkers for microglia and astrocytes, respectively, has also been seen in the spinal cord and DRG following CC [100]. Further, treatment with C021 can reduce the expression of IBA1, which suggests that C021 prevents the activation of microglia [100]. C021 treatment has also been shown to reduce the expression of the pronociceptive chemokines IL-1β and IL-18 in the spinal cord [100]. The role of CCR4 in pain is further supported by the effect of C021 on opioid efficacy. The analgesic effects of morphine and buprenorphine on CCI-induced tactile and thermal hypersensitivity are significantly enhanced by pre-treatment with C021 [99, 100, 109]. C021 also inhibits the development of morphine tolerance. C021 represents an emerging therapeutic which may aid in pain treatment and the prevention of opioid dependency [99].

### The Role of CCL17 and CCL22 in Post-Surgical Pain

CCL17/22 have been identified as key mediators of postoperative inflammatory pain [2]. Treatment with C021 before surgery reduces mechanical hypersensitivity, and post-surgery treatment alleviates mechanical and thermal hypersensitivity [2]. Using transcriptomic analysis of peripheral neurons from naïve mice, Silva et al. (2022) identified weak CCR4 expression on sensory neurons [2]. Blockade of CCR4 expressed on sensory neurons using small interfering RNA prevented pain responses to CCL22 administration and reduced mechanical hypersensitivity after an incisional wound [2]. Although no change in CCR4 expression was observed after the incisional wound, the excitability of CCR4+ sensory neurons was altered [2]. DRG neurons isolated from postoperative mice showed increased action potential (AP) frequency, and the application of CCL22 caused an additional increase in AP frequency [2]. This suggests CCL22 activation of CCR4 does alter the electrophysiological properties of sensory neurons and could be contributing to peripheral sensitization [2]. In support of this idea, Silva et al. (2022) found that neurons from mice treated with C021 or siRNA have no change in AP frequency when treated with CCL22 [2].

DCs in the skin, including Langerhans cells, have been identified as the source of CCL17/22 following incisional wounds. Mice depleted of DCs show reduced mechanical hypersensitivity after incisional wounds but no change in thermal hypersensitivity, and reduced CCL17/22 expression [2]. Collectively, the study suggests that an incisional wound induces upregulation of CCL17/22 from skin resident DCs, and that chemokines mediate inflammatory pain by directly acting on sensory neurons via CCR4 (Fig. 3) [2].

### Sex-Specific Differences in CCL17 and CCL22-Mediated Pain Pathways

Sex differences in CCL17- and CCL22-mediated pain pathways are increasingly evident in both animal and human studies [110]. In neuropathic pain-associated human dorsal root ganglia (DRG), females exhibit elevated levels of chemokines, including CCL17, compared to males [111]. This difference highlights a more substantial reliance on adaptive immune signalling in females, where CCL17 contributes to the activation of T cells in response to pain stimuli. By contrast, males predominantly engage microglial mechanisms, with limited involvement of chemokines such as CCL17 and CCL22 [111]. These findings underscore that females may experience heightened chemokine-driven immune responses to chronic pain conditions.

Preclinical studies in rodents also reveal pronounced sex-specific differences in the role of CCL17 and CCL22 [112]. Female rodents show greater activation of chemokine pathways mediated by CCR4, the receptor for CCL17, compared to males. This enhanced chemokine signalling aligns with findings that females rely more heavily on T cell-mediated pain pathways, which are significantly influenced by CCL17 and CCL22 [112]. These sex-specific mechanisms may result from hormonal regulation, as estrogen has been shown to modulate immune responses, potentially amplifying chemokine activity in females [112]. In contrast, males exhibit a reduced reliance on these chemokines, reflecting their preference for innate immune responses in pain signalling [112].

Clinical studies reinforce these sex differences, particularly in therapeutic contexts. For example, early-phase trials of anti-CCL17 therapies have primarily included male participants, and the lack of significant analgesic effects raises questions about the differential efficacy of these interventions across sexes [113]. Given the evidence of heightened CCL17 activity in females, these chemokines may be more relevant therapeutic targets for chronic pain in women [113]. The disparity in immune pathway engagement between sexes suggests the need for sex-specific approaches to pain management, particularly when targeting chemokine-mediated pathways.

## Potential Therapeutic Strategies Targeting the CCR4 Pathway

### Therapeutic Indications For CCR4-Anatagonists

Mogamulizumab is a humanised monoclonal antibody against CCR4 that the Federal Drug Agency approved for the treatment of relapsing and refractory mycosis fungoides and Sézary syndrome, two conditions associated with CTCL [114, 115]. In a clinical trial involving patients with CCR4-negative solid tumours, tumour regression in response to mogamulizumab was rarely observed [116]. This suggests that anti-CCR4 therapy directly targets malignant cells that express CCR4, thus enhancing antibody-dependent cellular cytotoxicity [114]. Additionally, mogamulizumab therapy can reduce the frequency of Tregs, resulting in limited associated immunosuppressive effects [117]. Also, it was found to reduce memory CD8+ T cells, which may impair the anti-cancer immune response [117]. Mogamulizumab has also been assessed in clinical trials for human T-lymphotropic virus type 1-associated myelopathy–tropical spastic paraparesis [118]. Results included improved muscle tone and a decrease in motor disability [118].

Another CCR4 antagonist, GSK2239633, was developed with the intent to treat asthma, but was not pursued because of low bioavailability in healthy subjects and its inability to inhibit CCR4 [119]. AZD-2098 and AZD-1678 were also identified as potent CCR4 antagonists. However, no clinical trials have assessed their efficacy in disease treatment [120]. However, there is no current evidence to support the use of any of these agents in reducing pain. However, their promising initial results in treating other clinical conditions may lead to their adoption in the context of reducing the inflammatory process associated with pain pathogenesis.

### Pharmacological Agents Targeting Chemokines For Pain Treatment

In addition to mogamulizumab, the only drugs targeting chemokine receptors that are approved for clinical use are maraviroc and plerixafor. Maraviroc is a CCR5 antagonist used for human immunodeficiency virus (HIV) treatment [121], and plerixafor is a CXCR4 inhibitor used to mobilize hematopoietic stem cells for autologous transplant in patients with non-Hodgkin’s lymphoma [122]. Consistent with the role of CCR5 and CXCR4 in pain, recent studies have shown that both drugs are capable of attenuating neuropathic pain responses in murine models [123, 124]. The analgesic effects of maraviroc and plerixafor have not been investigated in humans.

Despite the evidence of numerous chemokines contributing to pain responses, only CNTX-6970 and AZD2423, small-molecule antagonists for CCR2, have been investigated in clinical trials for pain treatment. CNTX-6970 is in an ongoing phase 2 trial for the treatment of osteoarthritic knee pain [125]. A phase 2 trial for AZD2423 in patients with posttraumatic neuralgia found no significant difference between AZD2423 and the placebo in average pain scores [126]. No other drugs intended for pain treatment have targeted chemokines or chemokine receptors.

Recent advancements support the potential of targeting CCL17 in pain management. GSK3858279, a novel high-affinity human monoclonal antibody (mAb) that inhibits CCL17, has shown promise in clinical trials for osteoarthritis (OA) knee pain [127]. In a phase I, randomised, double-blind, placebo-controlled study, GSK3858279 demonstrated significant reductions in average and worst knee pain intensity compared to placebo after 8 weeks of treatment [127]. These findings highlight the therapeutic potential of selectively targeting CCL17 in pain management, reinforcing the concept that distinct chemokine pathways may be critical in addressing specific pain responses.

The lack of therapeutic agents targeting chemokines may partly be attributed to the complexity of the chemokine system, with most receptors binding multiple ligands and expressed on numerous cell types [128]. This makes it difficult to predict the effects of targeting chemokines, as they are likely involved in multiple biological processes, and other chemokines may be able to compensate for their absence. Concerning CCR4, while CCL17/22 activate the same binding site, their contributions to disease pathogenesis and pain responses are not entirely equivalent. This suggests that therapeutics may need to specifically target either CCL17 or CCL22, while maintaining the function of the other [128].

The numerous allosteric binding sites on CCR4 may be beneficial to drug design, as they allow for improved specificity [55]. Additionally, the distinct effects on receptor internalization seen in response to CCL17/22 suggest they activate distinct downstream pathways that could be individually targeted [55].

However, it should be taken into consideration that developing a therapeutic agent for pain could have potential unintended effects on the immune system and systems influenced by immune cell activity, given the role of CCR4 activation in T cell migration. For instance, mice given a CCR4 antagonist showed worsened atherosclerosis in response to decreased Treg accumulation in plaques [129]. Specifically targeting CCR4 expressed on sensory neurons may mitigate this possibility but could be difficult due to the suspected low expression on sensory neurons compared to T cells [2].

## Conclusion

Chemokine signalling plays an important role in the development of pain responses, making it a possible target for therapeutic strategies aiming to relieve pain. CCL17, CCL22, and their receptor, CCR4, have specifically been linked to the process of pain signalling. CCL17 and CCL22 typically contribute to pain pathogenesis by mediating T cell chemotaxis. However, activation of CCR4 can directly and indirectly mediate pain responses. Future therapies should aim at targeting CCL17, CCL22, or CCR4 for attenuating the inflammatory process involved in pain pathogenesis.

## Data Availability

No datasets were generated or analysed during the current study.

## Appendix

### Methods

The PubMed database was searched for studies investigating CCL17 and CCL22 in pain responses using the search strategy: (CCL17 OR TARC OR CCL22 OR CCR4 OR “macrophage derived chemokine”) AND pain. The search identified 53 results, and 19 were included. Inclusion criteria: peer-reviewed study, written in English, assessing pain response or correlation of chemokines to pain levels, no conflict of interest. Additional searches were performed for sections on the neuroimmune modulation of pain, chemokines in pain, CCL17 and CCL22 in asthma, atopic dermatitis, and cancer, and clinical implications. Key words included nociceptors, pain, chemokine, signal, regulate, CXCL1, CXCL10, CXCL12, CXCL13, CCL2, CCL3, CCL17, CCL22, CX3CL1, TARC, “macrophage derived chemokine”, CCR4.

## Abbreviations

AP: Action potential
ATLL: Adult T cell leukemia/lymphoma
BDNF: Brain-derived Neurotrophic Factor
CCL: C–C motif chemokine ligand
CGRP: Calcitonin gene-related peptide
CNS: Central nervous system
CHO: Chinese Hamster Ovary
CCI: Chronic constriction injury
CFA: Complete Freund’s adjuvant
CTCL: Cutaneous T cell lymphomas
COX-2: Cyclooxygenase-2
DC: Dendritic cells
DRG: Dorsal root ganglion
ERK: Extracellular signal-related kinase
GM-CSF: Granulocyte–macrophage colony-stimulating factor
IFN-γ: Interferon- γ
IL: Interleukin
JMJD3: Jumonji domain-containing protein D3
LPS: Lipopolysaccharide
MDC: Macrophage-derived chemokine
MAPK: Mitogen-activated protein kinase
NGF: Nerve growth factor
PNI: Peripheral nerve injury
PACAP: Pituitary adenylate cyclase-activating polypeptide
si-RNA: Small Interfering RNA
SNL: Spinal Nerve Ligation
SP: Substance P
TARC: Thymus and activation regulated chemokine
TLR4: Toll Like Receptor 4
TGF-β: Transforming growth factor β
TNF: Tumour necrosis factor
Th2: Type 2 helper T cells
VIP: Vasoactive intestinal peptide
